# Long-Term Follow-Up of HPV16-Positive Women: Persistence of the Same Genetic Variant and Low Prevalence of Variant Co-Infections

**DOI:** 10.1371/journal.pone.0080382

**Published:** 2013-11-11

**Authors:** Daan T. Geraets, Leen-Jan van Doorn, Bernhard Kleter, Brigitte Colau, Diane M. Harper, Wim G. V. Quint

**Affiliations:** 1 DDL Diagnostic Laboratory, Rijswijk, The Netherlands; 2 GlaxoSmithKline Vaccines, Rixensart, Belgium; 3 Norris Cotton Cancer Center, Dartmouth Medical School, Hanover, New Hampshire, United States of America; University of Alabama at Birmingham, United States of America

## Abstract

HPV16 variants correlate with geographic origin and ethnicity. The association between infection with a specific variant and the cervical disease risk remains unclear. We studied the prevalence, persistence and association with cervical intraepithelial neoplasia (CIN) of different HPV16 variants, using cervical swabs and whole tissue sections (WTS) of biopsies from 548 women in the placebo group of a HPV16/18 vaccine trial. In HPV16-positive samples, HPV16 variants were identified by a reverse hybridization assay (RHA). Laser-capture micro-dissection (LCM) was performed for localized detection of HPV. HPV16 variants were determined in 47 women. Frequency of mixed HPV16 variant infections was lower (8.5%) than for multiple HPV genotypes (39.1%). Among 35 women having consecutive HPV16 variant-positive swabs, 32 (91.4%) had the same variant while in three (8.6%) women a change in variant(s) was observed. HPV16-positive WTS were obtained from 12 women having consecutive HPV16 variant-positive swabs. The same variant was present in WTS of 10 women, while two were negative. WTS of five women were histologically normal. A single HPV16 variant was detected in four women having CIN1-3, while additional HPV genotypes were found in three other women having CIN2 and CIN3. In the WTS of one woman with mixed genotypes, the HPV16 variant was assigned to a CIN2 lesion by LCM. HPV16 variant infections can be effectively studied in cervical swabs and tissue specimens by the HPV16 variant RHA. Multiple HPV16 variants in one woman are rare. The HPV16 genotype consistently detected in follow-up samples usually involves a persistent infection with the same variant.

## Introduction

Human papillomavirus (HPV) is a DNA virus that infects cutaneous and mucosal epithelium and induces epithelial proliferation. More than 40 HPV genotypes have been detected in the anogenital region, and clinically most important are the oncogenic (high-risk) HPV genotypes (e.g., HPV16 and HPV18) which are involved in the development of high grade cervical intraepithelial neoplasias (CIN) and cervical cancer [1-4]. HPV DNA has been detected in virtually all cervical cancer tissues [5], and persistent infection with an oncogenic HPV type, particularly HPV16 or 18, is recognized as the necessary cause of cervical cancer [6]. It is estimated that cervical cancer contributes to approximately 250,000 deaths and 500,000 new cases per year [7]. Vaccination against the most common oncogenic HPV genotypes, HPV16 and HPV 18, could prevent persistent infections of those genotypes and ultimately also prevent the development of up to 70% of cervical cancers worldwide [8,9].

Diagnosis of HPV infections is based on detection of its genomic DNA in cervical cell samples or cervical biopsy specimens by molecular methods, such as liquid hybridization (e.g., the Hybrid Capture 2 assay, Qiagen) [10,11] or the polymerase chain reaction (PCR) [12,13]. Liquid hybridization detects HPV DNA by direct probe hybridization and can distinguish between groups of high-risk and low-risk HPV genotypes, but does not permit identification of individual genotypes [14]. PCR methods amplify parts of the HPV DNA genome, resulting in a high analytical sensitivity and specificity.

HPV comprises at least 189 distinct genotypes [15]. By definition, DNA sequences in the L1 region of each pair of genotypes differ at least 10%. Each genotype comprises a number of subtypes showing between 2% and 10% nucleotide sequence difference. Within subtypes, variants exist, with a maximum sequence divergence of 2%.

HPV16 has been studied more extensively than other genotypes, and most information about viral variants is available from the E6, L1 and LCR regions of the viral genome [16-20]. The available information on other types, such as HPV 18, 31, 33, 35, 52, and 58 is limited [21], whereas information on the remaining HPV genotypes is virtually absent.

HPV16 subtypes and variants have been found to correlate with geographic origin and ethnicity of the infected patients. Sequence variation in the E6 region permitted discrimination between several subtypes, which have been designated as European prototype (E-P), African (with Af1 and Af2 as variants), Asian (As), North American (NA) and Asian American (AA) [19,20,22]. The prevalence of these subtypes and variants shows considerable geographic variation, which may have important epidemiological implications. 

Several studies have investigated the clinical and epidemiological relevance of HPV subtypes and variants. The association between infection with a specific subtype or variant of HPV (mainly HPV16) and the risk of cervical disease remains unclear. Some studies have reported a higher risk for specific variants, whereas other studies did not find any significant association [23-37]. In some populations, unique variants have been observed, but most studies were too limited to determine their clinical relevance. 

The aim of the present study was to identify the different variants of HPV16 in a large group of women participating in a HPV16/18 vaccine efficacy study who were assigned to the placebo group. The prevalence, persistence and association with cervical intraepithelial neoplasia (CIN) by different HPV16 variants were investigated in follow-up cervical swab and tissue material from the placebo group.

## Materials and Methods

### Clinical samples

HPV16-positive cervical swab and tissue specimens were selected for HPV16 variant analysis. These materials were available from the placebo group of women participating in a previous randomized efficacy trial of an HPV16/18 AS04-adjuvanted virus-like particle vaccine (*Cervarix*®, GlaxoSmithKline Vaccines, Rixensart, Belgium) [38]. Only materials from women in the placebo group were selected to rule out influence of the vaccine on the natural history of HPV16 variant infections. The protocol of this randomized controlled trial is described in detail in prior references [38,39]. Briefly, women had been recruited for this efficacy study through advertisements or previous participation in an HPV cross-sectional epidemiology study that took place between July and December, 2000. Investigators recruited women at study sites in North America (55%; Canada and USA) and Brazil (45%). Demographic descriptors were 69% White, 7% Black, 1% Asian, and 22% Other [38,39].

Women who had entered the vaccine trial were aged 15–25 years and were cytologically negative, seronegative for HPV16 and 18 antibodies by ELISA, and HPV-DNA-negative by PCR for 14 oncogenic HPV types (16, 18, 31, 33, 35, 39, 45, 51, 52, 56, 58, 59, 66, and 68) no more than 90 days before study entry. Health-care providers obtained cervical swab specimens for cytology and HPV DNA testing at screening and months 6, 12, and 18. At months 0 and 6, and subsequently every 3 months, women self-obtained cervicovaginal samples with two sequential swabs for HPV DNA testing, for up to 27 months [38]. Cervical swab specimens were collected in ThinPrep PreservCyt medium (Cytyc, Cytyc Corporation, Boxborough, MA, USA) and cytologically graded according to the Bethesda 2001 classification system. For those women whose cytology was abnormal, standard clinical protocols for colposcopy and biopsy were applied for clinical management, as described in detail previously [38,39]. Biopsy samples were formalin-fixed and paraffin-embedded (FFPE) and histologically classified by the CIN (cervical intraepithelial neoplasia) nomenclature grades 1, 2 and 3, where carcinoma-in situ was considered CIN3.

**Figure 1:**
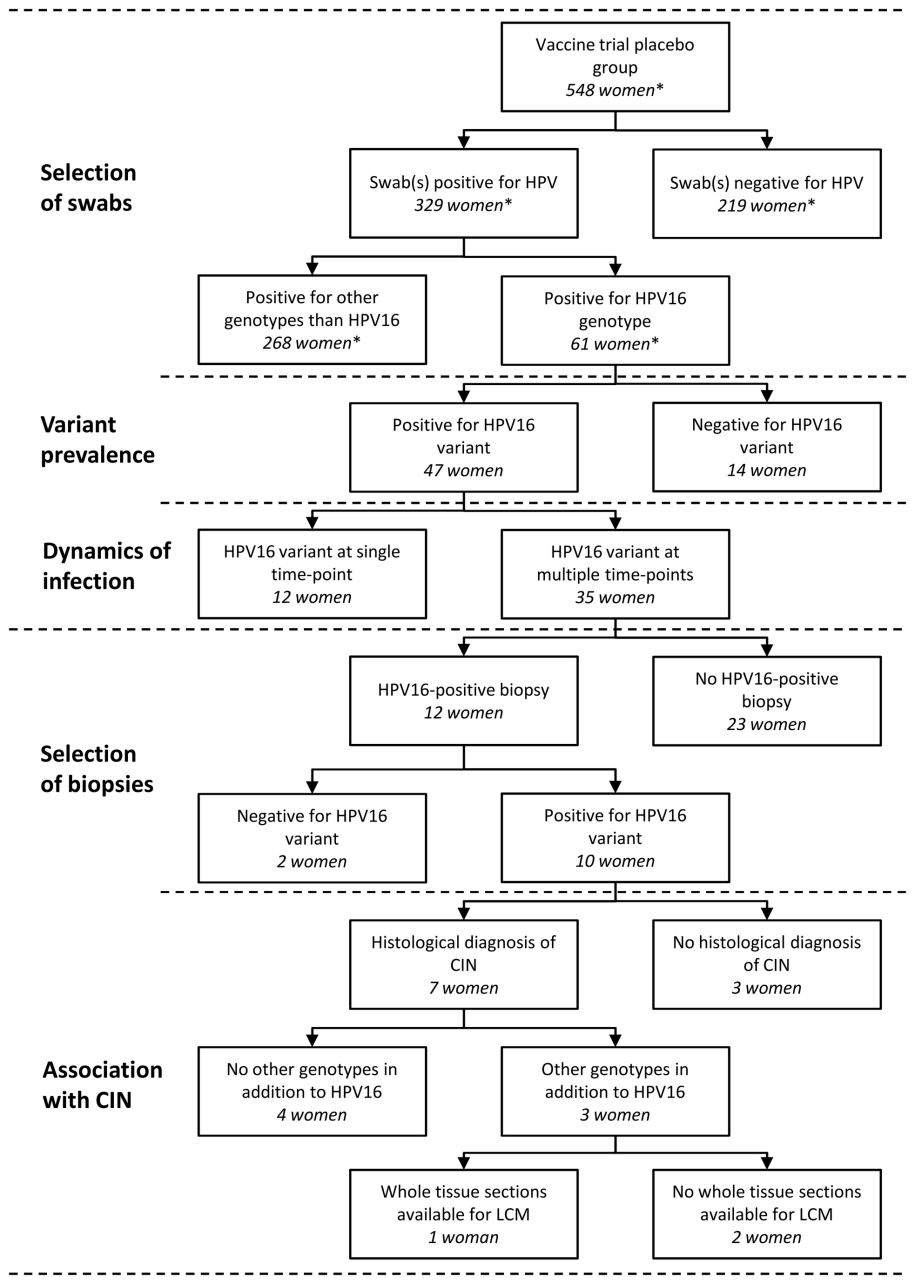
Flowchart of selection and subsequent analysis of clinical materials for presence of HPV16 variants. * This study included 5,144 cervical swab samples from 548 women assigned to the placebo group of a HPV16/18 vaccine trial, which is a subset of the total amount of clinical materials tested during this trial.

At the start of the current study for HPV16 variants, a subset of 5,144 cervical swab samples from 548 women assigned to the placebo group [38] had been tested so far for presence of HPV genotypes by SPF10 PCR-DEIA-LiPA25 (version 1; manufactured by LBP BV, The Netherlands, based on SPF10 technology licensed by Innogenetics). It should be noted that this is only a subset of the total amount of clinical materials analysed for HPV in the vaccine trial. Subsequently, those women in the placebo group whose cervical swabs and biopsy specimens were HPV16-positive (n=61), were selected for HPV16 variant analysis. A flowchart visualizing intake and subsequent analysis of clinical materials is shown in Figure 1.

### Ethics statement

The protocol, consent forms and amendments were approved by the central review board, as well as 32 institutional review boards at study sites in Canada, USA and Brazil, as already described by Harper et al [38,39]. Women signed separate written consents for study participation and colposcopy. All consents provided approval and consent for use of biologic materials for HPV analysis. For those under 18 years, parental written consent and assent from the participant were obligatory. The publicly available data is listed at http://clinicaltrials.gov under study numbers NCT00689741 and NCT00120848. 

### DNA isolation from cervical swabs and whole tissue sections

DNA was isolated from 200 μl of cervical swabs in PreservCyt suspension by the MagNA Pure LC instrument (Roche Diagnostics, Almere, the Netherlands), using the MagNA Pure LC Total Nucleic Acid isolation kit (Roche Diagnostics) according to the manufacturer’s instructions. DNA was eluted in 100 μl of elution buffer and 10 µl was used for each PCR reaction. Cervical tissue materials were processed according to a standardized sandwich method [40]. Briefly, twelve consecutive whole tissue sections (WTS) were obtained per FFPE block. Sections 1 and 12 were transferred onto glass slides and stained by hematoxylin and eosin (H&E, before and after). Sections 2, 3 and 4 were collected together in an Eppendorf tube for HPV PCR analysis (sections 5, 6 and 7 were stored in a back-up Eppendorf tube). Relatively large tissue sections were macro-dissected into smaller sections and processed separately for HPV analysis. Sections 8 and 9 were collected on glass slides and stained for p16 (sections 10 and 11 were collected as back-up slides). 

The stained slides were used for histopathological assessment by an expert pathologist. The three intermediate sections were digested overnight by 100 µl of proteinase K lysis buffer at 56°C. Proteinase K was heat-inactivated for 20 minutes at 95 °C. Ten µl of extracted DNA was used for each PCR reaction. If necessary, extracted DNA was diluted ten times prior to PCR to circumvent inhibition of amplification.

### Laser-capture micro-dissection (LCM) from whole tissue sections

Cervical tissue from one woman was processed by LCM technology for localized HPV analysis. The H&E- and p16-stained whole tissue sections (WTS) were scanned using digital microscopy (Aperio Technologies Inc, Vista, CA, USA). Separate, small regions of (borderline) CIN1 and CIN2 lesions as well as normal cervical epithelium were assigned by a pathologist, generally in the first WTS stained by H&E (Section 1). At least one region was selected per lesion grade, obtaining at least 5% of the complete lesion area. The selected areas were excised using the Zeiss P.A.L.M. microbeam ultraviolet (UV) laser microdissection and catapulting system and transferred to an AdhesiveCap500 opaque tube (Zeiss) [40]. LCM was performed on negative control tissue (human placenta section) prior to examination of each cervical tissue section. DNA was isolated from the LCM samples as described for WTS. 

### General detection of HPV DNA

Cervical swab and (micro-)dissected tissue samples were tested by the SPF_10_ PCR primer set to amplify a broad spectrum of HPV genotypes, as described earlier [41,42]. Briefly, this primer set amplifies a small fragment of 65 bp from the L1 region of HPV. Reverse primers contain a biotin label at the 5’ end, enabling capture of the reverse strand onto streptavidin-coated microtiter plates. Captured amplimers are denatured by alkaline treatment, and the captured strand is detected by a defined cocktail of digoxigenin-labelled probes, detecting a broad spectrum of HPV genotypes. This method is designated HPV DNA enzyme immunoassay (DEIA), providing an optical density value. The same SPF_10_ amplimers were used to identify the HPV genotype by reverse hybridization to the LiPA_25_ genotyping strip (version 1, manufactured by Labo Bio-medical Products, Rijswijk, the Netherlands, based on SPF10 technology licensed by Innogenetics). This line probe assay contains probes for 25 different HPV genotypes, i.e. HPV 6, 11, 16, 18, 31, 33, 34, 35, 39, 40, 42, 43, 44, 45, 51, 52, 53, 54, 56, 58, 59, 66, 68/73, 70 and 74. 

### PCR amplification and reverse hybridization analysis of HPV16 variants

Identification of the HPV variant in HPV16-positive swabs was performed using the E6-based HPV16 variant reverse hybridization assay (prototype research assay, Labo Bio-medical Products, Rijswijk, The Netherlands), as described previously [43]. The HPV16 reverse hybridization assay can be used on cervical swab samples with PCR primers amplifying a single region of approximately 570 bp, or by primer sets generating four overlapping fragments that are suitable for FFPE biopsy material. PCR conditions were slightly different in the current study. The MgCl_2_ concentration was increased from 2 mM to 2.5 mM, as well as the amount of each primer from 15 to 20 pmol. Furthermore, the annealing temperature was raised from 52 to 55 °C. The amplimers were cleaned-up by ExoSAP-IT reagent and analysed by reverse hybridization on the HPV16 variant strip according to the manufacturer’s instructions.

### Statistical analysis

 The baseline prevalence and distribution of HPV16 variants and presence of mixed variant infections was determined in the first HPV16-positive event of each woman, regardless if the sample was self-obtained or collected by the health-care provider. Three definitions for persistence of HPV16 variant infection detected at consecutive time-points were applied. The HPV16 variant infection was considered persistent if the same variant was detected in cervical samples over a minimum of 10 months (12-month definition), 5 months (6-month definition) or 2.5 months (3-month definition).

## Results

### Selection of HPV16-positive cervical swabs from placebo group (n=61 women)

Only materials from women in the placebo group were selected to rule out influence of the vaccine on the natural history of HPV16 variant infections. At the start of the current study, 5,144 cervical samples from women in the placebo group (n=548) [38] had been analysed so far by the HPV testing and genotyping algorithm. This comprises only a subset of the total amount of clinical materials collected and analysed in the complete vaccine trial. 

In this subset of 5,144 swabs from the total study population, 1,595 (31.0%) samples were found HPV-positive by SPF_10_ PCR/DEIA, and were genotyped by LiPA25. The most prevalent genotypes among HPV-positive swabs were HPV52 (15.4%), 16 (13.5%), 51 (11.5%), 53 (9.5%) and 66 (7.8%). Among the LiPA25-positive samples, 60.9% contained a single genotype, and the remaining 39.1% contained 2 to 7 HPV genotypes.

Subsequently, we selected 61 women from the placebo group who had at least one HPV16-positive cervical swab during the study period. A total of 207 HPV16-positive swab specimens were available for variant analysis. 

### HPV16 variant distribution in baseline cervical swabs (n=47 women)

Among the 207 HPV16-positive swabs from 61 women, 136 (65.7%) were positive and 71 (34.3%) were negative by the HPV16 variant RHA. In total, 47 women tested positive for a HPV16 variant in one or more swabs. In fourteen women, the variant could not be identified in any of their HPV16-positive swabs. All these women had been HPV16-positive by LiPA25 at only a single time point during follow-up. This suggests that these women experience transient HPV16 infections with low viral loads, below the detection limit of the HPV16 variant RHA.

**Figure 2:**
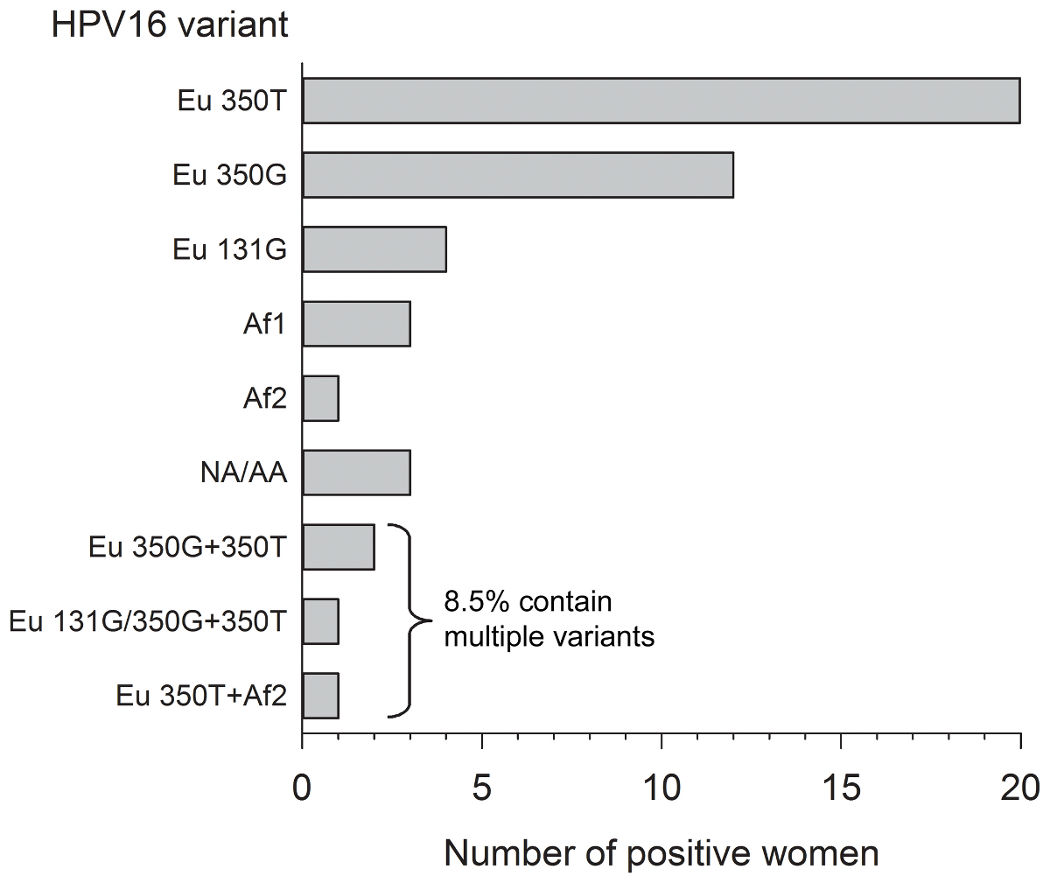
The distribution of HPV16 variants in the swabs of 47 women in the placebo group.

The distribution of HPV16 variants found at baseline among 47 women is shown in Figure 2. Of these women, only 4/47 (8.5%) demonstrated presence of a mixture of variants. This low proportion (8.5%) of samples containing multiple HPV16 variants is in contrast with the much higher frequency of samples (39.1%) containing multiple HPV genotypes.

Among the women with a single HPV16 variant (91.5%), European variants were the most prevalent (76.6%). African (8.5%) and North American/Asian-American variants (6.4%) were less common in this population.

### Dynamics of HPV16 variants in follow-up cervical swabs (n=35 women)

Among 35 out of the 47 women that were positive for a HPV16 variant (74.5%), the variant was identified at multiple time points during follow-up. In this group, the dynamics of the HPV16 variant infection was investigated over time. The women in this group had an average total of 10.4 swabs (range 4-13) per individual, obtained during an average follow-up period of 23.1 months (range 9-27) at 3-6 month intervals.

In 32 of the 35 women (91.4%), no HPV16 variant changes were observed throughout the follow-up period. Among these 32 women, variant ‘persistence’ was observed for at least 12 months in 10 women (31.3%), for 6 months in 14 women (43.8%) and for 3 months in 7 women (21.9%). In one woman (3.1%), the interval between two HPV16 Eu 350T-positive swabs was over 12 months, but without any HPV16-positive swabs in between. Thirty women (93.8%) were infected with a single HPV16 variant and two women (6.2%) had multiple variants. 

In 3 women, a change in HPV16 variants was observed during follow-up. In 2 of 35 women (5.7%), a mixture of HPV16 variants was observed in the first positive sample, and the composition of this mixture changed over time. A mixed infection with two European variants (Eu 350T and Eu 350G) progressed towards a single infection (Eu 350T). In the second woman, a mixed infection of Eu 350T and Af2 resolved to a single infection (Af2). In addition, 1 of 35 women (2.9%) was initially positive for Eu 350G and cleared this infection. After a HPV16-negative period of six months she was positive for a different European variant, Eu 131G. 

### HPV16 positive cervical tissue specimens (n=12 women)

From 12 women in the placebo group who had a HPV16 variant identified in a cervical swab, cervical tissue specimens were also collected. All specimens tested positive for HPV16 by SPF_10_ LiPA_25_ and were on average obtained 22 months after the baseline swab (range 8-39 months). For eight women, tissue specimens were collected between two HPV16 variant-positive swabs. From the remaining four women the tissue sample was taken after the last available HPV16 variant-positive swab. 

In 10 of 12 women (83.3%), the HPV16 variant was identified in the processed whole tissue section (WTS), i.e. Eu 131G (n=4), Eu 350T (n=4), and Eu 350G (n=2), while two women (16.7%) remained negative by the HPV16 variant RHA. In all cases, the variant identified in the WTS matched the variant found in the swabs taken before, and -if available- after the biopsy was obtained.

### HPV16 variant persistence and association with CIN (n=7 women)

The HPV16-positive WTS of the 12 women had been histopathologically examined for the worst lesion according to the CIN (cervical intraepithelial neoplasia) classification system. Five individuals had normal cervical tissue and the specific HPV16 variant, i.e., Eu 131G, could be determined in three of them. Seven women were diagnosed with lesions graded as either CIN1 (n=1), CIN2 (n=4) and CIN3 (n=2). 

For 4 of the 7 women with a CIN lesion, the WTS was single positive for HPV16, i.e., Eu 350T (n=3) and Eu 350G. No other HPV genotypes were detected by the LiPA25 algorithm. This strongly suggests that HPV16 most likely caused the lesion in these women. The other three women had at least one other HPV type in addition to HPV16 (Eu 350T, Eu 350G and Eu 131G) in the WTS (Table 1). 

**Table 1:** Results of HPV genotyping and HPV16 variant analysis performed on whole tissue sections of cervical tissue from seven women with diagnosed cervical intraepithelial neoplasia (CIN) graded as 1, 2, or 3.

**#**	**Histology**	**HPV16 variant**	**Other HPV genotypes**
1^a^	CIN1	Eu 350T	none
2	CIN2	Eu 350T	none
3	CIN2	Eu 350G	none
4	CIN3	Eu 350T	none
5	CIN2	Eu 350G	HPV58
6	CIN2	Eu 350T	HPV52
7^b^	CIN3	Eu 131G	HPV18 and 39

^a^Persistence of HPV16 variant Eu 350T in this woman is presented as a case in Table 2.

^b^Cervical whole tissue sections from this woman were further analyzed by laser-capture micro-dissection (LCM) and presented as a case in Table 3 and Figure 3.

An example of persistence of the same HPV16 infection is shown in Table 2. This demonstrates that the same HPV16 variant Eu 350T was consistently present (for at least 12 months) in follow-up swabs of a woman. She also had a HPV16 (Eu 350T) single positive tissue specimen of the cervix (with a diagnosed CIN1 lesion) taken in-between swabs.

**Table 2:** Case of persistent infection with HPV16 variant Eu 350T in a woman diagnosed with CIN1 (cervical intraepithelial neoplasia grade 1).

**Sample type**	**Observations**	**Month**								
		0	6	9	12	15	18	18.5	21	24
swab	HPV16 variant	neg	neg	neg	HPV16-Eu 350T	HPV16-Eu 350T	HPV16-Eu 350T		HPV16-Eu 350T	HPV16-Eu 350T
	Other HPV types	neg	neg	neg	neg	neg	neg		neg	neg
tissue	HPV16 variant							HPV16-Eu 350T		
	Other HPV types							neg		
	Histology							CIN1		

### HPV16 variant persistence and association with CIN investigated by LCM (n=1woman)

The tissue sections of one of these three women were available for localized HPV analysis by LCM technology, in an attempt to assign a specific HPV type to the lesion. Cervical tissue (CIN3) of this woman had been embedded in four blocks. Each block was processed by the sandwich method and macro-dissected into a total of 11 areas for HPV analysis. HPV16 (Eu 131G), 18, and 39 were detected in these areas, often as multiple infections. Three macro-dissected tissue sections could be examined by LCM. 

**Figure 3:**
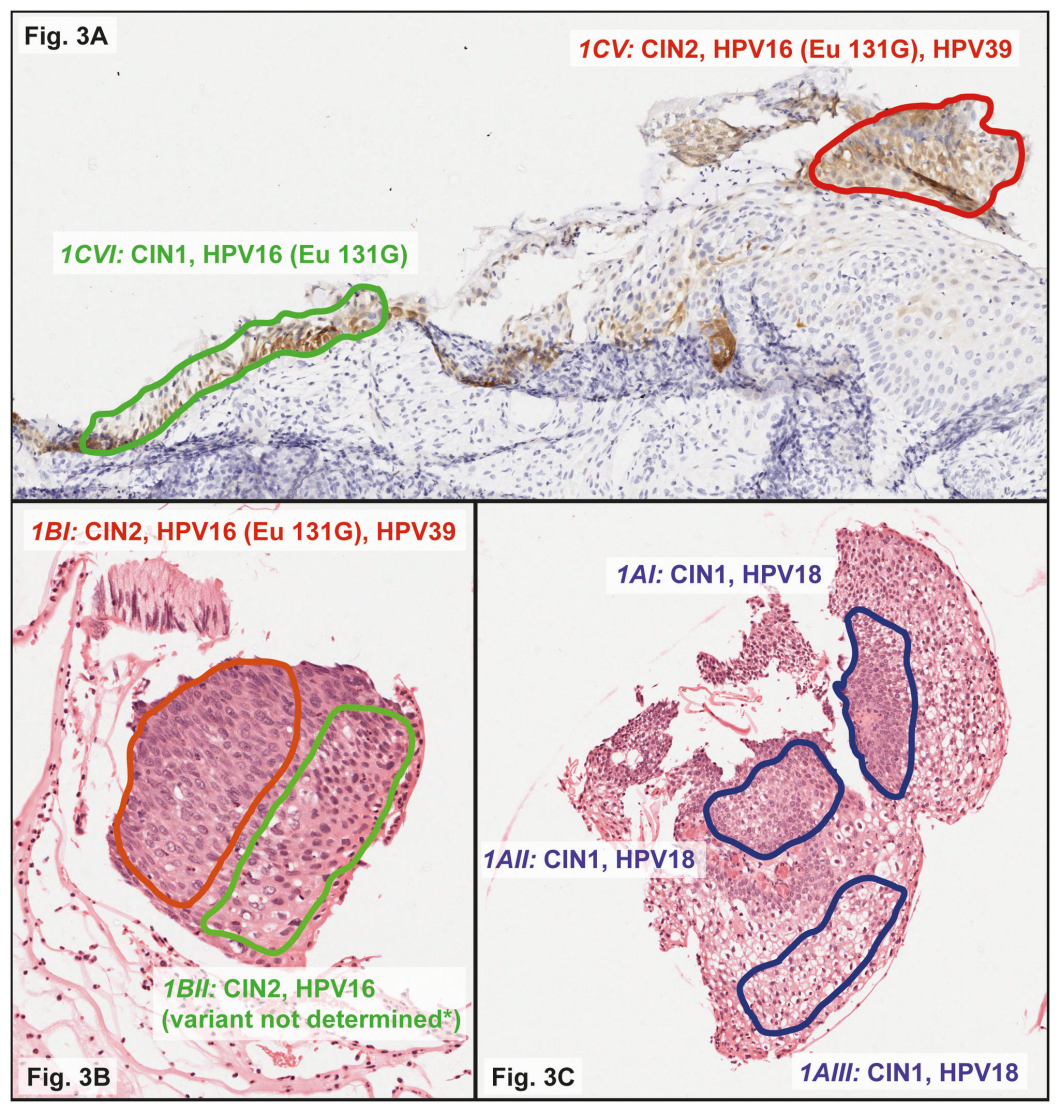
Histological images of localized HPV analysis by laser-capture micro-dissection (LCM) on a whole tissue section (WTS) positive for HPV16 (Eu 131G), 18 and 39. Local excision by LCM was performed on colored regions of different grades of cervical intraepithelial neoplasia (CIN), i.e., 1CV and 1CVI from the p16-stained section (Figure 3A), and 1BI, 1BII (Figure 3B), 1AI, 1AII, and 1AIII (Figure 3C) from the hematoxylin and eosin (H&E)-stained section. Excised regions were separately analyzed by LiPA25 and subsequently by the HPV16 variant RHA if HPV16-positive. All Images have been captured by ScanScope XT digital scanner (Aperio Technologies Inc, Vista, Ca, USA). * Region 1BII was positive by LiPA25 for HPV16 but negative by the HPV16 variant RHA.

In total, 11 regions were assigned by a pathologist, excised by LCM, and analysed for HPV by the LiPA25 algorithm (summarized in Table 3). HPV18 was found in LCM regions graded as CIN1. HPV16 was mostly detected in areas of CIN1/CIN2, either single or together with HPV39. This could indicate development of CIN2 lesions of different clonal origin that are colliding, as visualized in Figure 3. The five HPV16-positive LCM regions were analysed by the HPV16 variant RHA. The Eu 131G variant was identified in 3/5 (60.0%) regions, the others were negative.

**Table 3:** Case of whole tissue section with histologically diagnosed cervical intraepithelial neoplasia grade 3 (CIN3; worst lesion), in which multiple HPV genotypes were present.

**Block**	**Analyses on whole tissue sections**			**Analyses on LCM regions**		
	**Macro-dissected region**	**Histology**	**HPV type**	**Laser-capture micro-dissected region^a^**	**Histology**	**HPV type**
1	1A	CIN2	16, 18	1AI^b^	CIN1	18
				1AII^b^	CIN1	18
				1AIII^b^	CIN1	18
	1B	CIN2	16, 39	1BI	CIN2	16, 39
				1BII	CIN2	16
	1C	CIN1	16, 39	1CI	borderline CIN1	16
				1CII	borderline CIN1	negative
				1CIII	borderline CIN1	negative
				1CIV	normal	negative
				1CV^c^	CIN2	16, 39
				1CVI^d^	CIN1	16
	1D	normal	negative	No LCM	No LCM	No LCM
2	2A	CIN3	16	No LCM	No LCM	No LCM
	2B	CIN3	39	No LCM	No LCM	No LCM
3	3A	CIN3	16, 18	No LCM	No LCM	No LCM
	3B	CIN3	16	No LCM	No LCM	No LCM
	3C	CIN3/CIN2^e^	16, 18	No LCM	No LCM	No LCM
4	4A	CIN2	16, 39	No LCM	No LCM	No LCM
	4B	CIN3	16	No LCM	No LCM	No LCM

Localized detection of HPV was performed on laser-capture micro-dissected (LCM) regions from the hematoxylin and eosin (H&E) before section.

^a^Histological images of micro-dissected regions 1AI, 1AII, 1AIII, 1BI, 1BII, 1CV and 1CVI are shown in Figure 3.

^b^These regions were micro-dissected from the hematoxylin and eosin (H&E) after section instead of the H&E before section.

^c^Region 1CV was micro-dissected from the p16 section. This region corresponds with region 1CI, that was micro-dissected from the H&E before section.

^d^Region 1CVI was micro-dissected from the p16 section. This region corresponds with region 1CIII, that was micro-dissected from the H&E before section.

^e^Histological diagnosis was CIN3 in the H&E before section and CIN2 in the H&E after section

## Discussion

The present study describes HPV16 variant prevalence, persistence and association with cervical intraepithelial neoplasia (CIN) in HPV16-positive women, who were assigned to the placebo group of an HPV16/18 vaccine trial. The previously evaluated HPV16 variant RHA [43] proved to be a reliable and standardized method for cervical swabs, whole tissue sections and laser-capture micro-dissected tissue regions. 

Remarkably, the frequency of HPV16 positive swabs containing multiple HPV16 variants was very low, and this is in contrast to the frequency of samples containing multiple genotypes. The reason for this is unknown, but one could speculate that infection with a certain genotype is protective against super- or co-infection with another variant of that same genotype. Also, other studies investigating HPV variants reported surprisingly few mixed variant infections [44-46]. This may be partly due to the sensitivity and specificity of the methods used to identify variants. It is known that Sanger sequence analysis is an insensitive method to detect minority variants, with a sensitivity of approximately 25% for minority species. Despite the fact that reverse hybridization is very sensitive to detect such minority variants, the frequency was very low. Use of alternative methods, such as ultra deep sequencing, could detect multiple variants with even higher sensitivity, but we expect that the number of samples containing multiple variants of the same HPV genotype will remain low. For example, Swan et al used pyrosequencing on 97 HPV16-positive samples and multiple variants were found in only three specimens (3.1%) [47].

European HPV16 variants were highly prevalent in cervical swabs of our study population (76.6%), while African (8.5%) and North American/Asian-American variants (6.4%) were less common. In women with biopsy-confirmed CIN, only European variants were observed. The sample size of women with CIN is too small to investigate associations between specific variants and risk for CIN. The high prevalence of European HPV16 variants could be explained by the large proportion of women of European descent in this population, as suggested by the recruitment site (55% from USA and Canada, 45% from Brazil) and ethnicity of recruited women (69% White, 7% Black, 1% Asian, and 22% Other). Our findings are consistent with previous work by Zuna et al, who found high prevalence of European HPV16 variants (86%) among HPV16-positive women in the USA [46]. 

In most women, consecutive HPV16-positive swabs consistently contained the same variant. Variant changes were rarely observed in successive HPV16-positive swabs. In two women, a ‘persistent’ mixed HPV16 variant infection resolved to a single variant infection. Perhaps the immune response was able to clear one particular variant infection, but not the other. Another woman cleared a HPV16 variant but was infected with a different variant after six months. The primary infection did not induce a protective effect against the secondary infection, or perhaps the immune system was suppressed.

Data from our study show that most HPV16-positive swabs contain the same HPV16 variant over time, which provides further evidence for a persistent infection. From a group of women with multiple HPV16-positive swabs, also HPV16-positive cervical biopsy specimens were available. In 83.3% of these women, the same HPV16 variant was identified in swab samples as well as tissue specimens, which often contained CIN. The HPV16 variant was not identified in tissue samples of the other women (16.7%). This could indicate a slightly lower sensitivity of the HPV16 variant RHA compared to LiPA25 in FFPE tissue specimens. Although the number of samples analysed was small, these findings suggest that a HPV16 genotype consistently detected in follow-up cervical swabs and tissue samples usually involves a persistent infection with the same variant. A persistent infection with the same oncogenic HPV type, most notably HPV16, increases the risk for the development of (pre)cancerous lesions in the cervical epithelium, whereas re-infection with HPV16 after previous clearing probably is not associated with an increased risk.

Cervical tissues were further investigated if the persistent HPV16 variant infection was causative for development of CIN lesions. HPV16 was often found as the single genotype in whole-tissue sections of specimens with a diagnosed CIN lesion. This indicates that HPV16 was driving the lesion. In tissue of one woman, LCM technology was a suitable technique to assign causality of a CIN lesion to HPV16, when also other genotypes were present in the complete tissue sample. 

This study had limitations. Only a subset of clinical materials available from the previously described vaccine study group [38] was analysed. Our observations were based on a relatively small number of follow-up cervical swabs and tissue samples that was available to investigate prevalence and persistence of HPV16 variants. Moreover, the association between persistently present HPV16 variants and CIN could be studied in only seven women. The HPV16 variant RHA seems a suitable technique for different sample types, e.g., cervical swabs, whole tissue sections and laser-capture micro-dissected tissue regions. However, this should be confirmed in larger sample sizes.

In summary, the HPV16 variant-specific reverse hybridisation assay can be used to investigate prevalence of single and multiple HPV16 variants, to survey the dynamic behaviour of mixed variant infections in consecutive cervical swabs, to monitor persistence of a particular variant, and its association with cervical precancerous lesions. The HPV16 genotype consistently detected in follow-up cervical swabs and tissue samples usually involves a persistent infection with the same variant. 
